# Survival from cancer of the ovary in England and Wales up to 2001

**DOI:** 10.1038/sj.bjc.6604594

**Published:** 2008-09-23

**Authors:** H C Kitchener

**Affiliations:** 1 School of Cancer and Imaging Sciences, University of Manchester, Research Floor, St Mary's Hospital, Whitworth Park, Manchester, M13 0JH, UK

## Clinical presentation

Ovarian cancer is a disease of insidious onset without cardinal symptoms. Owing to this it tends to present late, by which time there are often widespread metastases throughout the peritoneal cavity. In some cases distension is the main symptom and in others the symptoms are pain, nausea and dyspepsia. Distension may be due solely to a large tumour, but quite frequently, ascites is also present. In some cases a large tumour remains encapsulated and confined to the ovary of origin; in other cases, relatively small-volume ovarian disease is associated with early metastases, particularly to the omentum with so-called ‘sago’ seedlings around the entire peritoneal surface. In a small minority of cases, breathlessness can be the presenting symptom because of pleural effusion secondary to peritoneal ascites.

## Diagnosis and treatment

Ovarian cancer is often diagnosable clinically by the detection of a large abdomino-pelvic mass with ascites and characteristic nodularity felt in the Pouch of Douglas anterior to the rectum. A careful history will often reveal no gastrointestinal symptoms, which makes a colonic or gastric tumour much less likely.

Over the past 15–20 years routine use of ultrasound has resulted in an unexpected diagnosis being made in women being investigated for vague abdominal pain and gastrointestinal symptoms. CA125, a monoclonal antibody detectable in serum, is usually elevated in ovarian cancer; almost invariably in cases of serous tumours but seldom in mucinous tumour. It is a valuable tumour marker and ovarian cancer is likely in cases of CA125 in excess of 1000 iu ml^−1^, with characteristic ultrasound findings of a solid/cystic mass in the pelvis and ascitic fluid. Certainty regarding the diagnosis is either made at laparotomy or by radiologically guided biopsy (e.g., Trucut).

Treatment of ovarian cancer involves a combination of surgery and chemotherapy, the latter being principally responsible for improved survival seen over the past 10 years. Surgery may be curative for the majority of early-stage cancers but these account for a small minority of cases overall. In many cases, surgery is an exercise in debulking, which has been considered worthwhile, particularly if the residual disease is confined to nodules of less than 1 cm. The importance of debulking has been debated for years. There is no doubt that maximally debulked women survive longer than those with bulk residual disease but what is unresolved is whether debulkable tumours are more chemosensitive and therefore, have a better prognosis. Nevertheless, ‘upfront’ primary surgery remains the international standard of care.

Chemotherapy using platinum is recommended in most cases, with the exception of stage 1a disease confined to the capsule. In recent years chemotherapy has been increasingly used before surgery in cases where women are ill, unfit for surgery and/or where the prospects for debulking seem to be poor. Ongoing trials will determine whether surgery ‘upfront’ or delayed after some chemotherapy is more effective.

## Interpretation of survival patterns

The overall improvement in 1-year survival between the mid 1980s and mid 1990s is probably because of increased access to platinum-based chemotherapy. In ovarian cancer, 1-year survival is dependant on optimal primary therapy. It is widely recognised that platinum chemotherapy is the most effective treatment for advanced disease. Carboplatin and taxol in combination will achieve response rates of 70%.

The improvement in 5-year survival is partly because of more widespread access to optimal primary treatment, but also reflects a more determined effort to re-treat recurrent disease. It has become clear that women who relapse more than 12 months following initial platinum-based chemotherapy may remain platinum sensitive and even further recurrences may be treated with other drug combinations.

Ovarian cancer is, therefore, becoming a more controllable chronic disease, although cure remains elusive in most cases. The rise in 5-year survival in some respects represents the fact of the United Kingdom catching up with the best practices in other countries. In the early 1990s insufficient specialist care meant that survival in ovarian cancer lagged behind other countries but that has been addressed with the Cancer Plan, and now women in the United Kingdom are treated by expert specialist teams. The rise in 10-year survival reflects what has been said above about treatment of recurrent disease and longer-term control.

## Deprivation gap

The deprivation gap at 1 year can probably be explained by a greater proportion of women in deprived communities having advanced disease and certainly in the past, poorer access to optimal treatment. This would have resulted in a higher proportion of treatment failures. Better access to specialist treatment has seen this gap close. The absence of a deprivation gap for 5-year survival is reassuring. It almost certainly reflects proportions of women with more chemosensitive tumours, with a longer time to relapse and higher response rates for recurrent disease. It probably also reflects improved access to specialist treatment and differences in comorbidity between the richer and poorer seem not to have an impact on survival.

## Overall comment

As ovarian cancer presents late, there has been a great deal of research into ovarian cancer screening. There is no pre-malignant phase, which can be recognised; but CA125 combined with ultrasound may be capable of detecting disease at an earlier, and hence more curable, stage. There is little evidence for this so far, but an ongoing very large randomised trial (UKCTOCS) will determine the effectiveness and cost effectiveness of ovarian cancer screening within 5 years.

As far as overall prognosis is concerned there is the prospect of continuing lengthening of survival, especially using newer targeted therapies. A recent clinical report from the NIH drew attention to increased survival with intraperitoneal chemotherapy, although the current regimens are rather toxic. A new strategy being evaluated in clinical trials, which could lengthen remission, is the use of biological drugs, such as anti-angiogeneic agents as maintenance therapy.

As the effectiveness of medical therapy increases the role of surgery may diminish, being used increasingly as a procedure to follow chemotherapy response rather than an initial procedure. Overall, incidence may fall as the ‘baby boomers’, who have used the oral contraceptive pill for many years, become postmenopausal and epidemiologically appear to be at reduced risk of ovarian cancer.

